# Enhancing Stoma Care Education for Junior Doctors: A Comprehensive Teaching Series

**DOI:** 10.7759/cureus.58014

**Published:** 2024-04-11

**Authors:** Akshay Bavikatte

**Affiliations:** 1 General and Colorectal Surgery, West Suffolk Hospital NHS Trust, Bury St Edmunds, GBR

**Keywords:** health education & awareness, small group teaching, surgical junior doctors, medical learning and teaching, advanced practice nurse, stoma care

## Abstract

Introduction

Colorectal stomas are prevalent in surgical wards and demand careful medical attention, particularly in stoma management. Junior doctors play a vital role in this care, but their limited exposure and training may hinder their ability, impacting patient care. Given the dearth of literature, we aimed to assess junior doctors' stoma care knowledge and the efficacy of a specialized teaching course in boosting their confidence and skills.

Methods

The research, conducted at the West Suffolk Hospital NHS Trust in the UK, engaged 60 junior doctors, predominantly from Foundation Year 1 and Year 2, from August 2021 to December 2022. To ensure effective management and assessment, participants were divided into four groups, each comprising 15 doctors. A pivotal aspect of the study was implementing a structured stoma teaching series delivered by a panel of seasoned surgical experts. This series, conducted every Friday for three weeks, comprehensively covered all facets of stoma care. Both before and after the teaching series, assessments were administered to measure the impact of this educational intervention on the participants' understanding of stomas. The study meticulously adhered to ethical guidelines, with all participants providing informed consent, and measures were implemented to guarantee anonymity, thus safeguarding the privacy and confidentiality of all individuals involved. The primary objective of this investigation was to evaluate the efficacy of the stoma teaching series in augmenting the knowledge and comprehension of stomas among junior doctors. The findings of this study hold significant potential in guiding healthcare professionals toward developing more efficacious stoma education programs, ultimately leading to improved patient care outcomes.

Results

The study involved 60 junior doctors categorized into four groups from August 2021 to December 2022. It aimed to assess their understanding of colorectal stomas, focusing on complications and their knowledge about stoma appliances and care nurses. A questionnaire was used to evaluate their knowledge in these areas at the start of their surgical rotation, which showed significant knowledge gaps among participants. Of the 60 participants, 48 (80%) expressed slight or no confidence in basic stoma care, while 54 (90%) admitted unfamiliar with managing stoma complications. Astonishingly, all 60 (100%) participants lacked awareness of fundamental stoma care concepts. Significant improvements were observed following a comprehensive stoma teaching series covering basic stoma knowledge, its complications and management, and practical stoma care. Feedback from the course revealed positive outcomes, with 54 (87%) doctors feeling confident or very confident in basic stoma knowledge and 48 (80%) reporting increased familiarity with managing stoma complications. Remarkably, all 60 (100%) doctors indicated comfort with stoma care concepts after the sessions. Participants emphasized the course's value in medical education and professional development, citing enhanced practical skills such as communication and teamwork.

Conclusion

Our study revealed junior doctors' limited stoma knowledge, emphasizing the need for a dedicated teaching program that significantly improves their understanding. Focused stoma education is vital for junior doctors to deliver optimal patient care, necessitating hospitals to promote awareness for improved patient outcomes.

## Introduction

The prevalence of patients with stomas in the United Kingdom is indeed increasing. It is estimated that approximately one in 500 individuals in the UK live with a stoma, with around 21,000 stoma formation surgeries performed annually [1]. Over 20,000 stomas are created each year in England to address various pathologies across all age groups [2]. Moreover, in the UK, there are approximately 102,000 ostomates, with roughly 21,000 stoma formation surgeries carried out yearly [3].

The education required for junior doctors to be knowledgeable about stoma care and management of stoma complications is lacking significantly. Studies have shown a gap in the training provided to junior doctors regarding stoma care [4,5]. Specifically, research indicates that newly qualified doctors may not be adequately prepared to care for patients with complex conditions, such as those requiring stoma management [6]. Furthermore, evidence suggests that poor knowledge and preparation contribute to errors made by junior doctors, emphasizing the need for focused education in specific areas like stoma care to enhance performance [7].

The study aims to assess junior doctors' knowledge and understanding of stoma care and evaluate the impact of a stoma teaching course on improving their knowledge and confidence in managing patients with stomas. The article will also provide participants feedback on the course's effectiveness.

## Materials and methods

The study was conducted at West Suffolk Hospital NHS Trust in the United Kingdom. It was designed to evaluate the level of knowledge about stomas among junior doctors, specifically, Foundation Year 1 (FY1) doctors who are newly graduated medical professionals undergoing supervised clinical training and Foundation Year 2 (FY2) doctors who build on this experience with increased responsibility and exposure to various specialties within the NHS. The study involved 60 participants who underwent surgical training from August 2021 to December 2022. The study aimed to assess the understanding of stomas within this cohort.

To ensure a comprehensive assessment, the participants were divided into groups of 15 doctors each, forming cohorts that underwent the study over the specified period. Each cohort spent four months in the surgical department, allowing for a consistent and structured evaluation of their knowledge and skills regarding stomas. As part of the study, all junior doctors were given a questionnaire to assess their knowledge of stomas. All study participants provided informed consent for the audit and publication of the results. Furthermore, the results have been presented anonymously to safeguard the privacy and confidentiality of all individuals involved.

Exclusion criteria

Doctors beyond FY2 and people who had already attended the course were excluded from the study.

Stoma teaching educational intervention

The stoma teaching series was implemented as part of the study aimed to enhance the participants' understanding of stomas through a structured and comprehensive educational program. This series spanned three weeks, with sessions held every Friday and delivered by various surgical experts.

During the teaching series, participants were exposed to diverse topics related to stomas, ensuring a holistic approach to their education. The teaching was also recorded to ensure access to the study population even if they could not attend due to various commitments. The series commenced with a session on the fundamentals of stomas, led by a colorectal surgical registrar. This portion likely focused on essential concepts such as stoma types, indications for stoma formation, and basic stoma care principles, providing participants with a solid foundation of knowledge. Subsequently, the teaching series delved into the complexities surrounding stoma complications with a colorectal consultant-led session. This segment likely explored potential complications associated with stomas, including but not limited to peristomal skin issues, prolapse, retraction, and herniation. By addressing these challenges, participants gained insights into recognizing, managing, and preventing complications, enhancing their clinical competency in stoma care. Finally, the series concluded with a session dedicated to stoma care facilitated by a specialized nurse. This section likely focused on practical aspects of stoma management, including stoma appliance selection, application techniques, peristomal skin care, and troubleshooting common issues. By leveraging the expertise of a stoma care nurse, participants received invaluable guidance on optimizing patient outcomes and promoting stoma well-being.

Following the completion of the teaching series, an assessment was conducted to evaluate the impact of the educational intervention on participants' knowledge of stomas. This assessment likely measured not only factual understanding but also the application of knowledge in clinical scenarios, thereby providing valuable feedback on the effectiveness of the teaching series in achieving its educational objectives. The study's objective was to evaluate how well the stoma teaching series improves junior doctors' knowledge and understanding of stomas, which is crucial for overall patient care.

## Results

Sixty junior doctors participated in the study, which was conducted from August 2021 to December 2022. The cohort was divided into four groups, each comprising 15 junior doctors, including FY1 and FY2 doctors. Each cohort spent four months in surgical rotation. The distribution of the various grades of junior doctors is presented in Table 1. 

**Table 1:** Distribution of grades of junior doctor n: number

Junior doctors grades	Number (60)
Foundation year 1 doctors (*n*, %)	38 (63.3%)
Foundation year 2 doctors (*n*, %)	22 (36.7%)

A questionnaire was administered at the start of their surgical rotation to assess the participants' understanding of the stomas. The questionnaire was divided into three main categories: basic understanding of stomas, stoma complications, and knowledge about stoma care consisting of awareness of the duties performed by stoma care nurses and practical aspects of stoma management. The questions within each category were carefully crafted to assess the participants' knowledge comprehensively. The basic understanding of the stomas category included questions about the basic anatomy and physiology and the different types of stomas. The stoma complications category included questions about understanding common complications that can arise after stoma surgery, such as blockages, leaks, and infections. The third category consists of awareness of stoma care nurses and practical aspects of stoma management, such as stoma siting and stoma bag change, as well as the role of stoma care nurses in managing stomas. The details of the questionnaire in each category are presented in Table 2.

**Table 2:** Stoma knowledge assessment questionnaire

Categories	Questions	Ratings from 1 to 5
Basics of Stoma	How confidently do you understand what is a stoma	1: Not confident
	I am aware of the indication of stoma	2: Somewhat confident
	I am aware of various different types of stoma	3: Neutral
	I am aware of differences between colostomy and Ileostomy	4: Confident
	I am aware of landmarks for stoma marking	5: Very confident
	Questions	Ratings from 1 to 5
Stoma complications	Are you familiar with complications of stoma	1: not familiar
	I can manage skin complication of stoma	2: somewhat Familiar
	I am aware of management of high output stoma	3 : Neutral
	I am aware of indication of emergency surgery in stoma	4: Familiar
		5: Very Familiar
	Questions	YES/NO
Stoma care	Are you aware of the stoma care nurses	YES/NO
	Are you aware of Duties of stoma care nurses	YES/NO
	Do you know how to change a stoma bag	YES/NO
	Are u aware of contents of a stoma care bag	YES/NO

**Figure 1:**
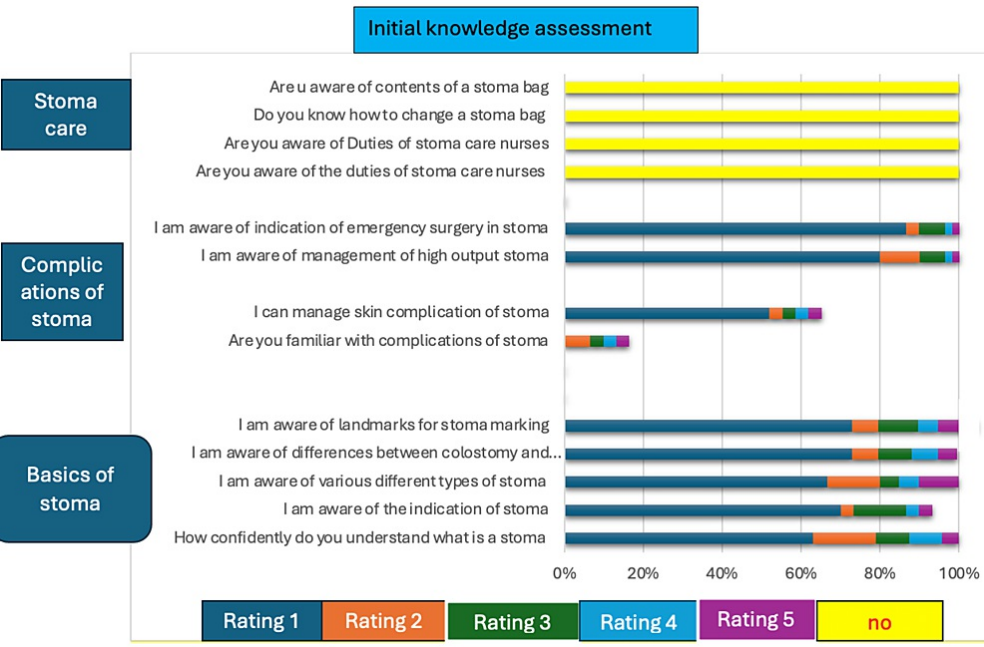
Graphical representation of Initial response before the stoma teaching course Basics of stoma: Rating - 1: not confident, 2: somewhat confident, 3: Neutral, 4: confident, 5: Very confident Complications of stoma: Rating - 1: not familiar, 2: somewhat familiar, 3: Neutral, 4: Familiar, 5: Very familiar

Regarding the responses, 48 (80%) out of 60 study group participants reported feeling either not confident or somewhat confident in their understanding of basic stoma care. This suggests a significant need for education and information on this topic. On the other hand, only six participants (10%) reported feeling confident in their understanding of basic stoma care, and no one reported feeling very confident. In terms of the management of stoma complications, 54 out of 60 participants (90%) reported feeling unfamiliar with the condition. This highlights the importance of providing healthcare professionals with the necessary training and resources to manage stoma complications effectively. Only six participants (10%) reported feeling familiar with managing stoma complications. Finally, regarding stoma care concepts, all 60 participants reported feeling unfamiliar with the role played by stoma care nurses and the contents of a stoma bag. This further emphasizes the need for comprehensive education and training on stoma care to improve patient outcomes and quality of care. These findings are presented in Table 3 and visually represented in Figure 1.

**Table 3:** Intial knowledge assessment prior to stoma teaching course n: Number

Categories		Ratings				
	Questions	1-Not confident	2- somewhat confident	3- neutral	4- confident	5- Very confident
	How confidently do you understand what a stoma is (n, %)	38 (64%)	10( 16%)	5 (8.4%)	5(8.4%)	2 (4.2%)
Basic of stoma	I am aware of the indication of stoma (n,%)	42 (70%)	6 (10%)	8 (13.3%)	2 (3.3%)	2 (3.3%)
	I am aware of the various types of stoma (n,%)	40 (66.7%)	8(13.3%)	3 (5%)	3 (5%)	6( 10%)
	I am aware of the differences between colostomy and ileostomy (n,%)	44 (73%)	4 (7%)	5 (8.4%)	4 (6.6%)	3 (5%)
	I am aware of the landmarks for stoma marking (n,%)	44 (73%)	4 (7%)	3 (5%)	3 (5%)	6 (10%)
		Ratings				
	Questions	1-Not Familiar	2- Somewhat familiar	3- Neutral	4- Familiar	5- Very Familiar
	Are you familiar with complications of stoma (n,%)	50 (83.3.%)	4 (6.7%)	2 (3.3%)	2 (3.3%)	2 (3.3%)
Stoma complications	I can manage skin complications of stoma (n,%)	52 (86.6%)	2 (3.4%)	2 (3.3%)	2 (3.3%)	2 (3.3%)
	I am aware of the management of high output stoma (n,%)	48 (80%)	6 (10%)	4 (6.6%)	1 (1.8%)	1 (1.8%)
	I am aware of the indication of emergency surgery in stoma (n,%)	52 (86.6%)	2 (3.4%)	4 (6.6%)	1 (1.8%)	1 (1.8%)
		Ratings				
	Questions	YES		NO		
	Are you aware of the duties of stoma care nurses (n, %)	0 (0%)		60(100%)		
Stoma care	Are you aware of the Duties of stoma care nurses(n, %)	0 (0%)		60 (100%)		
	Do you know how to change a stoma bag (n, %)	0 (0%)		60 (100%)		
	Are u aware of the contents of a stoma bag(n, %)	0 (0%)		60 (100%)		

Following the initial assessment, we implemented a comprehensive stoma teaching series for junior doctors. We scheduled the sessions every Friday during lunchtime over three weeks to ensure they could attend without conflicts. Attendance was diligently monitored and recorded in every session, and the sessions were also recorded so that the junior doctors could revisit the teachings at their convenience. The first session was delivered by a registrar in colorectal surgery, who provided an overview of the basics of stoma. They covered the anatomy of the stoma and its indications, ensuring that the junior doctors had a solid foundation of knowledge to build upon. The second session was led by a colorectal consultant, who delved deeper into managing stoma complications. They discussed the various challenges that may arise and provided practical advice on addressing them effectively. The third and final session was conducted by a stoma care specialist nurse, who focused on the practical aspects of stoma care. They covered the contents of a stoma bag and the process of stoma citing and demonstrated a practical demonstration of changing a stoma bag. This hands-on experience was invaluable for the junior doctors, as it allowed them to apply the knowledge they had gained in the previous sessions and develop their skills in stoma care.

Following the stoma teaching course, meticulously designed questionnaires were disseminated among the cohort of junior doctors to gauge their feedback meticulously. This approach facilitated a comprehensive evaluation of their perceptions regarding the course, providing valuable insights into their confidence level and proficiency in stoma care. The feedback was solicited from all 60 junior doctors who participated in the original audit, ensuring a representative sample for analysis.

Remarkably, the results revealed a highly positive response from the participants. A significant majority, comprising 52 out of 60 doctors (87%), expressed confidence or even high confidence in their grasp of basic stoma knowledge. Notably, not a single respondent rated their confidence level as low or not confident, underscoring the effectiveness of the course in instilling fundamental principles.

Moreover, a substantial proportion of the surveyed doctors, totaling 48 individuals (80%), indicated feeling comfortable in managing stoma complications, signifying a robust understanding and aptitude in handling complex scenarios. Although a minority of 6 doctors (10%) expressed neutrality in their confidence regarding stoma complications, the overall sentiment remains overwhelmingly positive.

**Figure 2:**
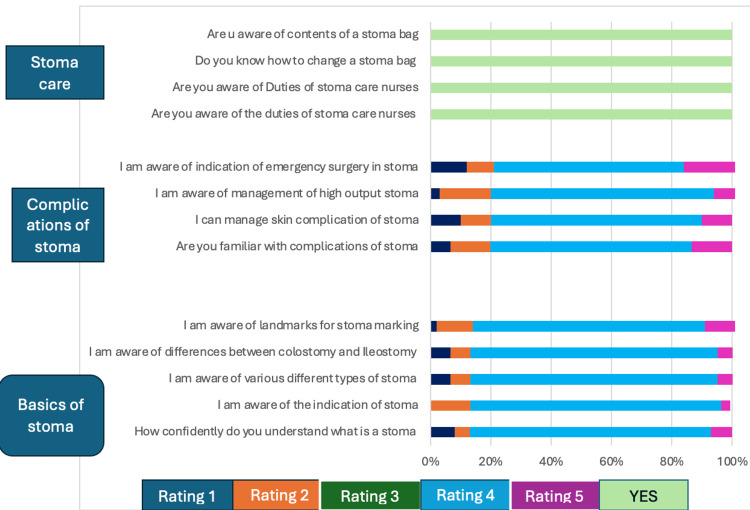
Graphical representation of feedback post stoma teaching course Basics of stoma: Ratings - 1: not confident, 2: somewhat confident, 3: Neutral, 4: confident, 5: Very confident Complications of stoma: Ratings - 1: not familiar, 2: somewhat familiar, 3: Neutral, 4: Familiar, 5: Very Familiar

Crucially, the comprehensive nature of the course is underscored by the unanimous sentiment among all 60 doctors (100%) who reported feeling comfortable and knowledgeable in stoma care. This resounding vote of confidence is further corroborated by the findings presented in Table 4 and visually depicted in Figure 2, which succinctly encapsulate the collective perception of the participants.

**Table 4:** Knowledge assessment post stoma teaching course n: Number

Categories		Ratings				
	Questions	1-Not confident	2- somewhat confident	3- neutral	4- confident	5- Very confident
	How confidently do you understand what a stoma is (n, %)	0 (0%)	5 (8%)	3 (5%)	48 (80%)	4 (7%)
Basic of stoma	I am aware of the indication of stoma (n,%)	0 (0%)	0 (0%)	8 (13.3%)	50 (84%)	2 (3%)
	I am aware of the various types of stoma (n,%)	0 (0%)	4 (6.6%)	4 (6.6%)	49 (82%)	3 (5%)
	I am aware of differences between colostomy and ileostomy (n,%)	0 (0%)	4 (6.6%)	4 (6.6%)	49 (82%)	3 (5%)
	I am aware of the landmarks for stoma marking (n,%)	0 (0%)	1 (2%)	7 (12%)	46 (77%)	6 (10%)
		Ratings				
	Questions	1-Not familiar	2- somewhat familiar	3- Neutral	4- familiar	5- Very familiar
	Are you familiar with complications of stoma (n,%)	0 (0%)	4(6.6%)	8 (13.3%)	40 (66.7%)	8 (13.3%)
Stoma complications	I can manage skin complications of stoma (n,%)	0(0%)	6 ((10%)	6 (10%)	42 (70%)	6 (10%)
	I am aware of management of high output stoma (n,%)	0(0%)	2 (3%)	10 (17%)	44 (74%)	4 (6%)
	I am aware of indication of emergency surgery in stoma (n,%)	0(0%)	7(12%)	5 (9%)	38 (63%)	10 (17%)
		Ratings				
	Questions	YES		NO		
	Are you aware of the duties of stoma care nurses (n, %)	60(100%)		0 (0%)		
Stoma care	Are you aware of the Duties of stoma care nurses(n, %)	60(100%)		0(0%)		
	Do you know how to change a stoma bag (n, %)	60(100%)		0(0%)		
	Are u aware of the contents of a stoma bag(n, %)	60(100%)		0 (0%)		

The junior doctors who participated in the teaching course found it highly beneficial and one of the most useful courses they had ever attended. They appreciated the well-structured format of the course and felt that it should be a part of the core learning curriculum for all medical students. The knowledge and skills gained from the course would be extremely valuable throughout their careers, regardless of the specialty they choose to pursue. The junior doctors were especially grateful for the practical hands-on experience and the opportunity to apply their knowledge in real-life scenarios. They felt this hands-on experience would be invaluable and stay with them throughout their careers. The course not only provided them with a solid foundation in medical knowledge but also helped them to develop important soft skills such as communication, teamwork, and critical thinking. The junior doctors were eager to apply what they had learned in the course to their daily work and felt that it would make them better equipped to handle the challenges of the medical field. Overall, the course significantly impacted the junior doctors' professional development, and they were grateful for the opportunity to participate.

## Discussion

Surgical stomas are frequently utilized in NHS surgical wards and hospitals for various gastrointestinal surgical procedures. These stomas are created for reasons such as abdominal penetrating trauma, enteric perforation, intestinal obstruction, and intestinal tuberculosis [8]. Common stomas include loop ileostomy, sigmoid colostomy, and transverse colostomy [9]. As the population ages and life expectancy increases, the prevalence of conditions necessitating stomas, such as rectal neoplasms and inflammatory bowel diseases, is expected to grow, leading to more household relatives with stomas. Understanding the impact of stomas on patients and their families is vital for delivering comprehensive care and support to this expanding population [10,11].

There is a notable deficiency in the existing literature regarding the knowledge of stomas among junior doctors, who possess an insufficient comprehension of this subject. Research has shown that the management of stomas, which can present with considerable complication rates and psychological consequences for patients, is frequently requested by junior doctors who may have limited experience in this area, ultimately resulting in suboptimal patient care [12]. In alignment with these findings, our evaluation revealed that most participants lacked confidence in their foundational grasp of stoma management, underscoring the pressing need for enhanced educational interventions to address this critical aspect of patient care.

Complications arising from stomas pose significant challenges to patients' well-being, necessitating meticulous management. These complications include prolapse, surgical site infections, necrosis, leakage, granuloma formation, retraction, stenosis, parastomal hernia, peristomal skin diseases, and dermatitis. They often result in physical discomfort, psychological distress, and financial burdens for affected individuals [13]. Healthcare professionals must remain vigilant in monitoring and addressing these complications to ensure optimal care and preserve patients' quality of life. Interestingly, research indicates that patients may possess a better knowledge of stoma care than medical professionals [14]. Similarly, our study also revealed a significant lack of knowledge concerning identifying and managing stoma complications among healthcare providers. This underscores the critical need for targeted educational efforts to enhance professionals' competency in patient care.

Examining current literature on medical education and interdisciplinary collaboration is crucial, given the knowledge gap among junior doctors in the UK regarding specialized nurses' roles, especially in stoma care. Davenport highlights junior doctors' lack of awareness about specialized nurses' contributions, hindering effective interdisciplinary collaboration and impacting patient outcomes [15]. Similarly, our study reveals that our cohort of junior physicians expresses a dearth of knowledge concerning stoma care nurses, their roles and responsibilities, and practical aspects of stoma care, such as stoma site selection, stoma bag changing procedures, and stoma content management. Addressing these knowledge gaps is imperative to foster enhanced interdisciplinary collaboration and improve patient care quality in stoma care settings.

The existing literature underscores the pressing need for educational interventions and training programs to address the knowledge gaps among junior doctors, particularly in areas such as stoma management, prescribing practices, emergency procedures, and various medical conditions. By enhancing the knowledge and skills of junior doctors, patient care and safety can be significantly improved [16]. Research on nurse-doctor co-teaching shows the benefits of collaborative teaching in boosting interprofessional collaboration. Junior doctors gain insight into specialized nurses' roles by fostering teamwork and respect between doctors and nurses. Addressing the knowledge gap among junior doctors in specialized nurses' roles, including stoma care nurses, requires educational initiatives promoting interdisciplinary collaboration and communication. This enhances awareness of specialized nurses' contributions, improving teamwork skills and patient care quality [17].

Our educational initiative embraces contemporary pedagogical methods, incorporating succinct yet dynamic sessions that blend short bursts of instruction with breaks for hands-on activities, moments of reflection, and the integration of multimedia elements. Extensive research, as highlighted by existing literature [18,19], underscores these strategies' efficacy in enhancing student engagement and long-term retention and application of knowledge. Moreover, Patel et al.'s findings shed light on the cognitive benefits of providing access to recorded lectures, emphasizing the importance of review materials in reinforcing learning outcomes [20]. Within our structured approach, the stoma teaching series unfolds as a weekly event during lunch hours, facilitated by appropriately qualified individuals, with supplementary recordings accessible to accommodate scheduling conflicts. The culminating session, led by experienced stoma care nurses, offered participants hands-on demonstrations and comprehensive explanations of stoma bag application techniques. The enthusiastic reception of these practical sessions underscores their invaluable contribution to enriching participants' understanding and proficiency in stoma care management.

Following the implementation of our educational initiative, we were delighted to witness an overwhelmingly positive response from the cohort. Most participants reported a significant increase in confidence regarding their understanding of stoma care, with many expressing a newfound familiarity with managing stoma-related complications. Notably, there was widespread acknowledgment of the invaluable services provided by stoma care nurses, with participants expressing newfound awareness and appreciation for their role in patient care. Furthermore, participants conveyed a heightened sense of competence in stoma bag application, indicating a tangible improvement in their practical skills. The overall feedback received for the course was exceptionally positive, underscoring the effectiveness and impact of our educational program in empowering healthcare professionals with essential knowledge and skills in stoma care management.

The main objective of our article is to draw attention to the lack of knowledge regarding stomas and their care among junior doctors, as there is a dearth of literature addressing this crucial issue. Stomas are an inevitable aspect of medical practice, irrespective of the specialization a doctor chooses, whether professional or personal. Our study's limitations include a small sample size and the absence of evidence demonstrating whether the education provided has improved patient care. However, our hospital has implemented a regular stoma teaching series for junior doctors delivered at the commencement of the surgical rotation. We hope other medical institutions will follow suit to enhance awareness and deliver better patient care.

## Conclusions

Our study underscored the insufficient understanding of stomas among junior doctors, underscoring the necessity for a specialized stoma teaching curriculum and its beneficial effects on enhancing their comprehension of stomas. Offering targeted education and resources centered on stomas is essential to guarantee that healthcare professionals are adequately informed and competent in delivering the best possible patient care. Hospitals should assume a proactive role in championing education and raising awareness among their staff, ultimately leading to enhanced patient care and outcomes.
